# Effectiveness of a Structured Educational Intervention on Healthcare Providers' Knowledge of Paediatric Dehydration Management: A Closed-Loop Clinical Audit at Almanagil Teaching Hospital, Sudan

**DOI:** 10.7759/cureus.114119

**Published:** 2026-08-07

**Authors:** Ethar Ali, Sara Mohamed Abdelmoteleb, Muzdlefa Idriss Ibrahim Ahmed, Razan Nabil Siddig Mohamed, Israa Hamza Abdelgader Musa, Ruaa Izeldin Mohamed Altyib, Abrar Elnour Ibrahim Ahmed, Narmen Hamadelnil Ibrahim Eltrafi, Nura Hassan Osman, Areeg Hussien Mohamed Mustafa, Hajir Jamal Salman Abdalgani, Tawasul Siefuddin Awadelkarim Mohamed, Reem Saifeldin Osman, Hana Arif Seedahmed Obeid, Rumaysaa Suliman Mahmoud Albashir, Amani Abdelazim Abdelrahman Abdelbaqi, Albashir Alkabashi Albashir Almsbah, Amin Omer Amin Ahmed, Mustafa Mohamed

**Affiliations:** 1 Faculty of Medicine, Ahfad University for Women, Omdurman, SDN; 2 Pediatrics, Almanagil Teaching Hospital, Almanagil, SDN; 3 Internal Medicine, Dongola Specialized Hospital, Dongola, SDN; 4 Neonatology, Maternity and Children Hospital, Najran, SAU; 5 Medicine, Hamad Medical Corporation, Doha, QAT; 6 General Medicine, Ministry of Public Health, Doha, QAT; 7 Internal Medicine, Al Neelain University, Khartoum, SDN

**Keywords:** clinical audit, educational intervention, evidence-based practice, healthcare providers, imci, integrated management of childhood illness, paediatric dehydration, quality improvement, sudan

## Abstract

Background

Accurate assessment and management of paediatric dehydration are fundamental to reducing preventable morbidity and mortality associated with acute diarrhoeal disease. The World Health Organization (WHO) Integrated Management of Childhood Illness (IMCI) guidelines provide standardized recommendations for dehydration assessment and treatment; however, variations in healthcare providers' knowledge may compromise adherence to these evidence-based standards. This audit evaluated the effectiveness of a structured educational intervention in improving healthcare providers' knowledge of paediatric dehydration management.

Methods

A closed-loop clinical audit was conducted in the Department of Paediatrics at Almanagil Teaching Hospital, Sudan. The first audit cycle was undertaken over two weeks (27 April-10 May 2026) and included 50 healthcare providers. Following identification of baseline deficiencies, a two-week educational programme based on the WHO IMCI guidelines was implemented (11 May-24 May 2026). A second audit cycle was conducted over one month (25 May-24 June 2026) involving 54 healthcare providers using the same standardized questionnaire. Knowledge regarding dehydration classification and WHO management Plans A, B, and C was assessed and compared between audit cycles.

Results

Significant improvements were observed across all evaluated domains following the educational intervention. Correct identification of severe dehydration increased from 23 (46.0%) to 46 (85.2%) (p* *< 0.001), while accurate classification of some dehydration improved from 30 (60.0%) to 51 (94.4%) (p* *< 0.001). Knowledge of appropriate management using WHO Plan A improved from 35 (70.0%) to 51 (94.4%) (p* *< 0.001), Plan B from 28 (56.0%) to 48 (88.9%) (p* *< 0.001), and Plan C from 42 (84.0%) to 53 (98.1%) (p* *= 0.010).

Conclusion

A structured educational intervention significantly improved healthcare providers' knowledge of paediatric dehydration management according to the WHO IMCI guidelines. Integrating regular educational programmes with repeated closed-loop clinical audits may represent an effective and sustainable strategy for strengthening adherence to evidence-based practice and promoting continuous quality improvement in paediatric healthcare.

## Introduction

Acute diarrhoeal disease remains a major cause of morbidity and mortality among children under five years of age worldwide despite substantial progress in preventive and therapeutic interventions. Dehydration secondary to diarrhoea continues to account for a considerable proportion of preventable childhood deaths, particularly in low- and middle-income countries where delayed recognition and inappropriate fluid management remain significant contributors to adverse clinical outcomes. Early assessment of dehydration severity and prompt initiation of appropriate rehydration therapy are therefore fundamental components of paediatric care and have played a pivotal role in reducing childhood mortality over recent decades. Oral rehydration therapy (ORT), together with continued feeding and appropriate zinc supplementation, is recognized as one of the most effective and cost-efficient public health interventions for preventing deaths associated with acute diarrhoeal illness. These advances have substantially improved survival; however, optimal outcomes continue to depend on timely clinical assessment and strict adherence to evidence-based management guidelines [1-3].

To standardize the management of childhood illnesses, the World Health Organization (WHO) and the United Nations Children's Fund (UNICEF) developed the Integrated Management of Childhood Illness (IMCI) strategy, an evidence-based approach that has been widely adopted in low- and middle-income countries to improve the quality of paediatric care. Within the IMCI framework, children with acute diarrhoea are clinically classified into three categories, i.e., no dehydration, some dehydration, and severe dehydration, based on specific clinical signs rather than laboratory investigations. This classification guides the implementation of standardized treatment algorithms (Plans A, B, and C), which emphasize appropriate oral or intravenous fluid therapy according to the severity of dehydration. The IMCI approach has been shown to facilitate rapid clinical decision-making, reduce unnecessary interventions, improve consistency of care, and contribute to better clinical outcomes when implemented correctly. Consequently, the WHO continues to recommend IMCI as a cornerstone of paediatric case management, particularly in resource-constrained healthcare settings, where timely recognition and appropriate treatment of dehydration are essential for reducing preventable morbidity and mortality [4,5].

Successful implementation of the WHO IMCI recommendations depends not only on the availability of standardized guidelines but also on healthcare providers' ability to accurately assess dehydration severity and apply the appropriate management plan in routine clinical practice. Accurate recognition of clinical signs, correct classification of dehydration, and timely initiation of the recommended treatment are essential to prevent progression to severe dehydration and its associated complications. Nevertheless, studies from low- and middle-income countries have consistently reported deficiencies in healthcare providers' knowledge and adherence to IMCI recommendations, including misclassification of dehydration severity, inappropriate selection of treatment plans, and errors in fluid management. These deficiencies may compromise patient safety, increase unnecessary hospital admissions and intravenous fluid use, and ultimately reduce the effectiveness of otherwise well-established evidence-based guidelines. Consequently, strengthening clinicians' knowledge and promoting adherence to standardized protocols have become key priorities for improving the quality and safety of paediatric care through continuous professional education and quality improvement initiatives [6].

In Sudan, acute diarrhoeal disease continues to contribute substantially to childhood morbidity, particularly in resource-limited healthcare settings where implementation of evidence-based management recommendations may be challenged by workforce and resource constraints. Although the IMCI strategy has been incorporated into paediatric healthcare services nationally, published evidence evaluating healthcare providers' knowledge of paediatric dehydration management in Sudanese hospitals remains scarce. This highlights the need for locally conducted quality improvement initiatives to identify knowledge gaps and strengthen adherence to evidence-based recommendations.

Clinical audit is a fundamental component of healthcare quality improvement that systematically evaluates current clinical practice against explicit evidence-based standards, implements targeted interventions to address identified deficiencies, and reassesses performance to determine whether meaningful improvements have been achieved. As a closed-loop quality improvement process, clinical audit promotes continuous monitoring of clinical performance, reinforces adherence to established guidelines, and supports sustainable improvements in patient care. Educational interventions delivered in response to identified practice gaps, including interactive teaching sessions, case-based discussions, feedback, and dissemination of clinical guidelines, have consistently demonstrated their effectiveness in improving healthcare providers' knowledge, adherence to recommended practices, and overall quality of care across a wide range of clinical settings. Consequently, combining structured education with closed-loop clinical audit represents an effective strategy for translating evidence-based recommendations into routine clinical practice and ensuring sustained improvements in healthcare delivery [7-9].

Against this background, this closed-loop clinical audit aimed to evaluate healthcare providers' knowledge and adherence to the WHO IMCI guidelines for the assessment and management of paediatric dehydration at Almanagil Teaching Hospital, Sudan. Specifically, the audit sought to identify baseline deficiencies in clinicians' knowledge, implement a structured educational intervention targeting the identified gaps, and determine the effectiveness of this intervention by comparing healthcare providers' performance before and after its implementation. Ultimately, the audit was conducted to promote adherence to evidence-based practice and support continuous quality improvement in the management of children presenting with dehydration.

## Materials and methods

Study design and setting

A closed-loop clinical audit was conducted in the Department of Pediatrics at Almanagil Teaching Hospital, Gezira State, Sudan, to evaluate healthcare providers' knowledge regarding the assessment and management of paediatric dehydration according to the WHO IMCI guidelines. The audit employed a two-cycle before-and-after design comprising a baseline assessment, implementation of targeted educational interventions, and a re-audit to evaluate the effectiveness of the interventions. The audit was conducted over approximately two months, from 27 April 2026 to 24 June 2026, as part of the hospital's continuous quality improvement programme.

The first audit cycle was conducted over two weeks, from 27 April to 10 May 2026, to establish baseline knowledge and identify deficiencies in adherence to the WHO IMCI recommendations. This was followed by a two-week educational intervention from 11 May to 24 May 2026, during which targeted teaching activities were implemented. The second audit cycle (re-audit) was subsequently conducted over one month, from 25 May to 24 June 2026, using the same methodology and assessment tool to determine the impact of the intervention.

Audit standards

The audit standards were derived from the WHO IMCI guidelines and the WHO Pocket Book of Hospital Care for Children, which provide internationally recognized evidence-based recommendations for the assessment and management of paediatric dehydration [1,3,4]. Healthcare providers' knowledge was evaluated against these standards, including recognition of dehydration severity, identification of danger signs, and appropriate implementation of WHO treatment Plans A, B, and C.

Participants

Healthcare providers working in the Department of Pediatrics at Almanagil Teaching Hospital were eligible to participate in the audit. Participants included house officers, medical officers, registrars, and specialists who were actively involved in the assessment and management of paediatric patients. All eligible clinicians present during each audit period were invited to participate. As this was a departmental clinical audit designed to evaluate adherence to predefined standards, no formal sample size calculation was performed. Instead, a total population sampling approach was adopted, whereby all eligible healthcare providers available during each audit period were invited to participate.

The first audit cycle included 50 healthcare providers, while the second audit cycle included 54 healthcare providers. As the audit evaluated departmental adherence to evidence-based standards rather than individual performance, participation by the same clinicians in both cycles was not required. Participation was voluntary, and all responses were anonymized before analysis to ensure confidentiality and minimize response bias. As participant identifiers were intentionally not collected, it was not possible to determine the number of clinicians who participated in both audit cycles. Nevertheless, both audit cycles included comparable professional groups from the same department, allowing assessment of departmental adherence to audit standards rather than individual performance.

Data collection tool

Data were collected using a structured self-administered questionnaire developed by the authors based on the WHO IMCI guidelines for paediatric dehydration management (Appendices). The questionnaire was reviewed by two senior consultant paediatricians for face and content validity to ensure that all items accurately reflected current WHO IMCI recommendations.

The questionnaire was specifically designed for this clinical audit and was not adapted or reproduced from any previously published questionnaire. It evaluated participants' knowledge across nine predefined audit domains derived from the WHO IMCI guidelines: (1) recognition of severe dehydration, (2) classification of some dehydration, (3) recognition of danger signs, (4) knowledge of oral rehydration solution (ORS), (5) knowledge of zinc supplementation, (6) appropriate indications and selection of intravenous fluids, (7) appropriate implementation of WHO Plan A, (8) appropriate implementation of WHO Plan B, and (9) appropriate implementation of WHO Plan C. The identical questionnaire was administered during both audit cycles to ensure direct comparison of performance before and after the intervention. As the questionnaire was developed specifically for this quality improvement audit, formal psychometric validation and test-retest reliability assessment were not undertaken.

First audit cycle

The baseline audit was conducted between 27 April and 10 May 2026. Participants completed the standardized questionnaire under standardized conditions, and responses were compared with predefined WHO IMCI standards. The baseline assessment identified important deficiencies in knowledge regarding dehydration classification and appropriate treatment selection, thereby informing the design of the subsequent two-week educational intervention.

Educational intervention

Following completion of the first audit cycle, a structured educational programme was implemented over two weeks (11 May-24 May 2026). The programme consisted of four interactive educational sessions, each lasting approximately 60 minutes, delivered by senior consultant paediatricians experienced in IMCI implementation. All sessions followed a standardized teaching plan to ensure that identical educational content was delivered to all participants. Attendance registers were maintained, and the majority of eligible healthcare providers attended at least one educational session. The intervention consisted of interactive educational sessions delivered by senior paediatric clinicians, focusing on the WHO IMCI guidelines for assessment and management of paediatric dehydration. Educational activities included lectures, case-based discussions, review of dehydration classification criteria, recognition of danger signs, and practical application of WHO treatment Plans A, B, and C. Educational summaries based on WHO guidance were also distributed to reinforce key learning objectives and encourage adherence to standardized clinical practice.

The educational intervention was implemented as a department-wide quality improvement initiative and was offered to all eligible healthcare providers. Consequently, no attempt was made to restrict dissemination of educational materials or exclude participants based on their involvement in the baseline audit, as the primary objective was to improve overall departmental adherence to WHO IMCI recommendations.

Second audit cycle

The second audit cycle was conducted between 25 May and 24 June 2026 using the same questionnaire, audit standards, and assessment methodology employed during the baseline audit. A total of 54 healthcare providers participated in the re-audit, allowing direct comparison of departmental knowledge before and after implementation of the educational intervention.

Outcome measures

The primary outcome was healthcare providers' knowledge of evidence-based paediatric dehydration management according to the WHO IMCI guidelines. Nine predefined audit criteria were assessed: (1) accurate identification of severe dehydration, (2) correct classification of some dehydration, (3) recognition of danger signs, (4) appropriate knowledge of ORS, (5) appropriate knowledge of zinc supplementation, (6) correct indications and selection of intravenous fluids, (7) appropriate implementation of WHO Plan A, (8) correct implementation of WHO Plan B oral rehydration therapy, and (9) appropriate implementation of WHO Plan C intravenous fluid management. Compliance with each audit criterion was expressed as the number and percentage of participants providing correct responses during each audit cycle.

Statistical analysis

Data were entered into Microsoft Excel (Microsoft Corporation, Redmond, WA) and analysed using SPSS version 24.0 (IBM Corp., Armonk, NY, USA). Categorical variables were summarized as frequencies and percentages. Comparisons between the first and second audit cycles were performed using the chi-square test or Fisher's exact test where appropriate. A two-sided p-value <0.05 was considered statistically significant.

Ethical considerations

The audit received approval from the Department of Pediatrics and the Administration of Almanagil Teaching Hospital before commencement and was conducted in accordance with institutional clinical audit governance procedures. Ethical approval was granted under IRB Reference Number PD-1998. Participation was voluntary, and written informed consent was obtained from all healthcare providers before questionnaire completion. Participant anonymity and confidentiality were maintained throughout the audit by removing all personal identifiers before data analysis. The project was conducted in accordance with the ethical principles of the Declaration of Helsinki and was designed solely as a quality improvement initiative to enhance adherence to the WHO IMCI guidelines in paediatric clinical practice.

## Results

A total of 104 healthcare providers participated across both audit cycles, comprising 50 participants in the first audit cycle and 54 participants in the second. Participant characteristics are presented in Table 1. In both audit cycles, medical officers and house officers constituted the majority of participants, with representation from registrars and specialists. Most participants worked in the paediatric ward or emergency department, and the distribution of years of clinical experience was broadly similar between the two audit cycles.

**Table 1:** Demographic characteristics of healthcare providers participating in the two audit cycles. Values are presented as frequency (n) and percentage (%). Percentages are calculated within each audit cycle.

Characteristic	First cycle (n = 50), n (%)	Second cycle (n = 54), n (%)
Professional grade		
House officer	18 (36.0)	20 (37.0)
Medical officer	16 (32.0)	17 (31.5)
Registrar	10 (20.0)	11 (20.4)
Specialist	6 (12.0)	6 (11.1)
Department		
Paediatric ward	24 (48.0)	26 (48.1)
Emergency department	16 (32.0)	17 (31.5)
Outpatient clinic	10 (20.0)	11 (20.4)
Years of clinical experience		
<1 year	12 (24.0)	13 (24.1)
1-3 years	18 (36.0)	20 (37.0)
4-6 years	11 (22.0)	12 (22.2)
>6 years	9 (18.0)	9 (16.7)

Following implementation of the educational intervention, significant improvements were observed across all evaluated domains of paediatric dehydration management (Table 2). Correct identification of severe dehydration increased from 23 (46.0%) during the first audit cycle to 46 (85.2%) in the second cycle (χ² = 17.85, p < 0.001). Similarly, accurate classification of some dehydration improved from 30 (60.0%) to 51 (94.4%) (χ² = 17.88, p < 0.001).

**Table 2:** Comparison of healthcare providers' knowledge of paediatric dehydration management before and after the educational intervention. Values are presented as frequency (percentage). Ninety-five percent confidence intervals (95% CI) for proportions were calculated using the Wilson method. Effect size is reported as the phi coefficient (φ), where values of approximately 0.10, 0.30, and 0.50 indicate small, moderate, and large effects, respectively. Comparisons between audit cycles were performed using Pearson's chi-square test. A two-sided p-value <0.05 was considered statistically significant. ORS: oral rehydration solution.

Audit criterion	First cycle (n = 50), n (%) 95% CI	Second cycle (n = 54), n (%) 95% CI	χ²	φ	p-value
Severe dehydration identification	23 (46.0) (33.0-59.6)	46 (85.2) (73.4-92.3)	17.85	0.414	<0.001
Some dehydration classification	30 (60.0) (46.2-72.4)	51 (94.4) (84.9-98.1)	17.88	0.415	<0.001
Plan A management	35 (70.0) (56.2-80.9)	51 (94.4) (84.9-98.1)	10.84	0.323	<0.001
Plan B management	28 (56.0) (42.3-68.8)	48 (88.9) (77.8-94.8)	14.27	0.370	<0.001
Plan C management	42 (84.0) (71.5-91.7)	53 (98.1) (90.2-99.7)	6.57	0.251	0.010
IV fluid indications and selection	29 (58.0) (44.2-70.6)	49 (90.7) (80.1-96.0)	14.84	0.378	<0.001
ORS knowledge	25 (50.0) (36.6-63.4)	46 (85.2) (73.4-92.3)	14.84	0.378	<0.001
Zinc supplementation	29 (58.0) (44.2-70.6)	48 (88.9) (77.8-94.8)	12.89	0.352	<0.001
Recognition of danger signs	30 (60.0) (46.2-72.4)	51 (94.4) (84.9-98.1)	17.88	0.415	<0.001

Knowledge of the WHO treatment protocols also improved significantly. Correct responses regarding Plan A management increased from 35 (70.0%) to 51 (94.4%) (χ² = 10.84, p < 0.001), while knowledge of Plan B management improved from 28 (56.0%) to 48 (88.9%) (χ² = 14.27, p < 0.001). Compliance with Plan C management was already relatively high at baseline but increased further from 42 (84.0%) to 53 (98.1%), representing a statistically significant improvement (χ² = 6.57, p = 0.010).

Significant improvements were also observed in other key areas of paediatric dehydration management. Correct knowledge regarding IV fluid indications and selection increased from 29 (58.0%) to 49 (90.7%) (χ² = 14.84, p < 0.001), ORS knowledge improved from 25 (50.0%) to 46 (85.2%) (χ² = 14.84, p < 0.001), zinc supplementation knowledge increased from 29 (58.0%) to 48 (88.9%) (χ² = 12.89, p < 0.001), and recognition of danger signs improved from 30 (60.0%) to 51 (94.4%) (χ² = 17.88, p < 0.001).

**Figure 1:**
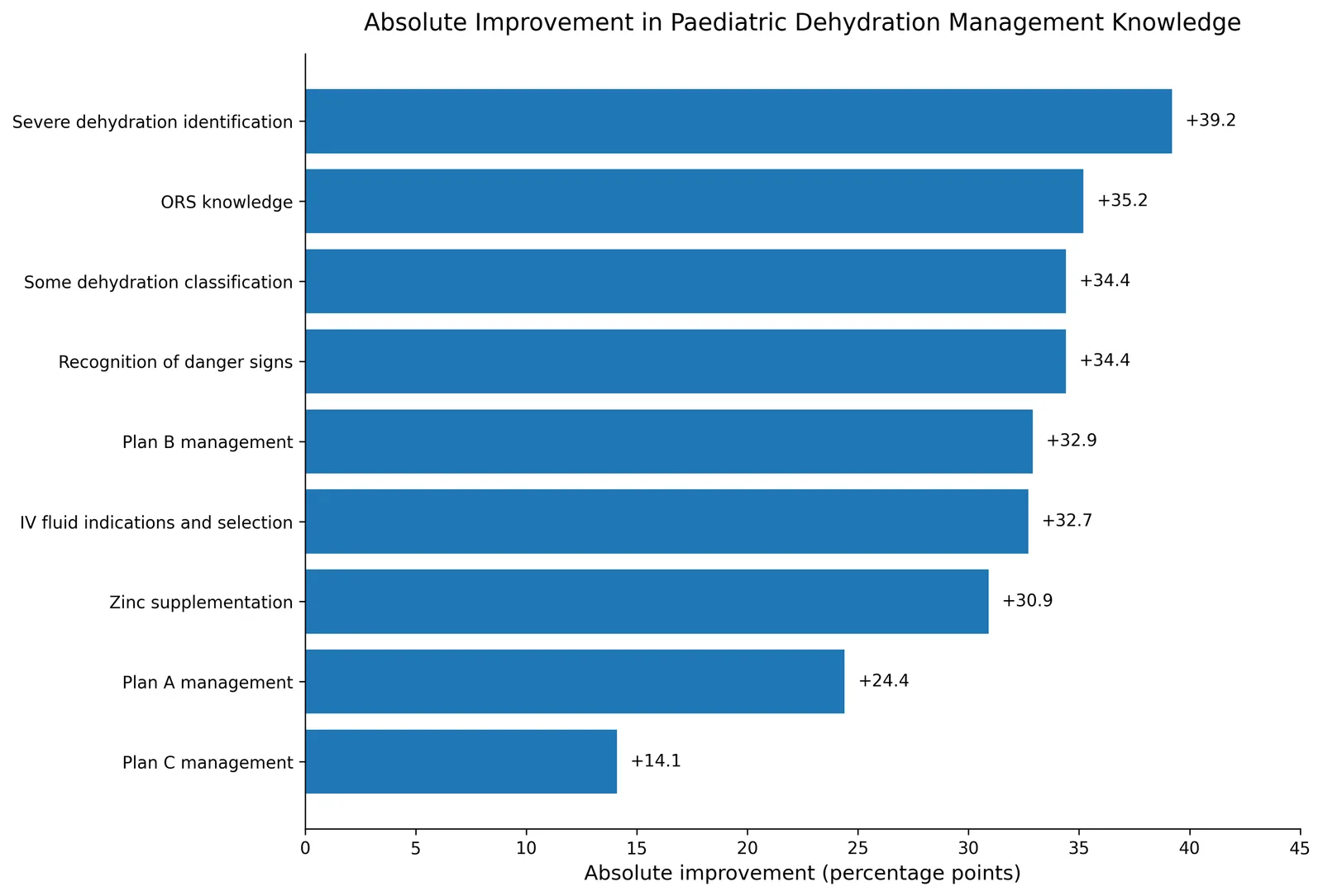
Absolute percentage-point improvement in healthcare providers’ knowledge of paediatric dehydration management following the structured educational intervention. ORS: oral rehydration solution.

Overall, all nine audit criteria demonstrated statistically significant improvements following the educational intervention. The largest absolute improvement was observed in the recognition of severe dehydration (+39.2%), followed by ORS knowledge (+35.2%), accurate classification of some dehydration (+34.4%), recognition of danger signs (+34.4%), IV fluid indications and selection (+32.7%), Plan B management (+32.9%), zinc supplementation (+30.9%), Plan A management (+24.4%), and Plan C management (+14.1%) (Figure 1).

## Discussion

The present closed-loop clinical audit demonstrated that a structured educational intervention was associated with significant improvements in healthcare providers' knowledge of paediatric dehydration management according to the WHO IMCI guidelines [4]. Statistically significant improvements were observed across all evaluated domains, including recognition of severe dehydration, classification of some dehydration, and appropriate application of WHO treatment Plans A, B, and C. These findings suggest that targeted educational strategies can effectively address baseline knowledge deficiencies and enhance adherence to standardized evidence-based recommendations.

The greatest improvement was observed in the recognition of severe dehydration, a finding of particular clinical importance because prompt identification and timely management of severe dehydration are essential to prevent hypovolaemic shock, organ dysfunction, and avoidable childhood mortality. Our findings support the growing body of evidence that continuous professional education and quality improvement initiatives play a pivotal role in strengthening clinicians' competence and promoting safer, more consistent paediatric practice. Although recent literature has increasingly focused on improving dehydration assessment and timely intervention, it continues to emphasize that accurate recognition of dehydration remains fundamental to improving patient outcomes and reducing preventable complications [6].

Our findings are consistent with previous quality improvement initiatives conducted in low- and middle-income countries, where structured educational interventions and implementation of IMCI have improved healthcare providers' adherence to evidence-based clinical guidelines. Previous studies from South Africa have demonstrated significant improvements in the assessment of danger signs, rational prescribing, and overall quality of paediatric care following IMCI implementation, while multicountry studies in sub-Saharan Africa have shown that periodic IMCI training and refresher programmes are associated with better adherence to recommended assessment and management practices. More recent evidence also suggests that although educational interventions substantially improve provider knowledge, sustained improvements in clinical practice require complementary system-level strategies to address the persistent "know-do" gap. Collectively, these findings support the role of structured educational interventions and closed-loop quality improvement initiatives as practical and scalable approaches for strengthening paediatric care in resource-constrained healthcare settings [10-12].

The improvements observed are likely attributable to the educational strategy employed during the intervention period. Rather than relying solely on dissemination of written guidelines, the intervention incorporated structured educational sessions that emphasized practical application of the WHO IMCI recommendations [4]. Interactive learning encourages active participation, facilitates clarification of misconceptions, and promotes integration of theoretical knowledge into clinical reasoning. Furthermore, presenting standardized assessment algorithms within clinically relevant scenarios enables healthcare providers to develop a systematic approach to evaluating dehydration and selecting the appropriate management plan. Such educational strategies are particularly valuable in busy clinical environments where rapid decision-making is essential and standardized approaches help minimize unwarranted variations in practice.

An important observation from this audit is that substantial improvements were achieved using relatively simple and low-cost quality improvement interventions. The educational programme required no additional equipment or sophisticated technology, yet it produced meaningful improvements in knowledge across all evaluated domains. This finding highlights the feasibility of implementing similar interventions in resource-limited healthcare settings, where financial and infrastructural constraints frequently limit access to advanced educational resources. Integrating focused educational activities into routine departmental teaching programmes may therefore represent a practical and sustainable approach for strengthening adherence to evidence-based clinical guidelines while optimizing the use of existing institutional resources [13].

Although the educational intervention produced significant short-term improvements, maintaining these gains requires continuous reinforcement. Knowledge acquired during educational sessions may decline over time if opportunities for revision and practical application are limited. Sustained improvement therefore depends on embedding evidence-based recommendations into routine clinical practice through regular continuing professional development activities, periodic refresher sessions, bedside teaching, simulation-based training, and ongoing supervision by senior clinicians. Incorporating repeated closed-loop clinical audits into departmental quality assurance programmes may also help identify emerging knowledge gaps, monitor long-term adherence to established standards, and ensure that improvements are sustained over time [14].

The findings of this audit also demonstrate the value of the closed-loop clinical audit methodology as a structured quality improvement process. By systematically identifying deficiencies, implementing targeted interventions, and reassessing performance using identical audit standards, the audit cycle provides objective evidence of improvement while promoting accountability and continuous professional development. Beyond evaluating individual knowledge, this approach encourages standardization of clinical practice, supports evidence-based decision-making, and fosters a culture of continuous quality improvement within healthcare institutions. Consequently, the methodology used in the present audit could be adapted to other paediatric clinical pathways and healthcare settings to improve compliance with evidence-based recommendations and strengthen patient safety [15].

Several strengths should be acknowledged. The audit employed internationally recognized WHO IMCI guidelines as predefined audit standards, ensuring that performance was evaluated against evidence-based recommendations [4]. The use of a closed-loop design allowed objective assessment of the effectiveness of the educational intervention, while application of the same assessment tool during both audit cycles facilitated direct comparison of knowledge before and after implementation. Furthermore, inclusion of healthcare providers from different professional grades enhances the relevance of the findings to routine paediatric clinical practice.

Sustaining improvements in guideline adherence also depends on strengthening the broader healthcare system. Continued availability of oral rehydration solution, intravenous fluids, zinc supplements, and essential monitoring equipment is necessary to enable clinicians to implement WHO IMCI recommendations effectively. In addition, adequate staffing levels, manageable workloads, and institutional support for continuing professional development are likely to enhance long-term adherence to standardized clinical practice. Within the local context, integrating IMCI refresher training into routine departmental teaching schedules and incorporating periodic closed-loop clinical audits into hospital quality assurance activities may represent practical and sustainable strategies for maintaining improvements over time.

Nevertheless, the audit has several limitations. It was conducted at a single teaching hospital, which may limit the generalizability of the findings to other healthcare settings. The assessment focused on healthcare providers' knowledge using a structured questionnaire rather than direct observation of clinical practice; therefore, improvements in knowledge may not necessarily translate into identical improvements in bedside performance. Furthermore, because the same questionnaire was administered during both audit cycles and the educational intervention was designed around the knowledge gaps identified during the baseline assessment, some of the observed improvement may have been influenced by participants' familiarity with the assessment items (testing effect) rather than solely reflecting a true improvement in knowledge. Additionally, the re-audit was conducted shortly after completion of the educational intervention, and the long-term retention of knowledge was not evaluated. Future multicentre studies incorporating direct assessment of clinical practice, independently validated assessment tools, and longer follow-up periods would provide a more comprehensive evaluation of the sustainability of educational interventions and their impact on patient outcomes.

## Conclusions

This closed-loop clinical audit demonstrated that a structured educational intervention substantially improved healthcare providers' knowledge of paediatric dehydration assessment and management according to the WHO IMCI guidelines. The findings support the integration of regular IMCI-focused educational sessions and periodic closed-loop clinical audits into routine quality improvement activities at Almanagil Teaching Hospital to reinforce evidence-based practice and sustain improvements in clinician knowledge. Given the minimal additional resources required to implement the intervention, similar educational programmes could be adapted and implemented in other departments and healthcare institutions across Sudan to strengthen adherence to the WHO IMCI recommendations and improve the quality of paediatric care. Future multicentre studies evaluating long-term knowledge retention, changes in clinical practice, and patient-related outcomes are warranted to further establish the impact, scalability, and sustainability of such interventions.

## Figures and Tables

**Figure 2:**
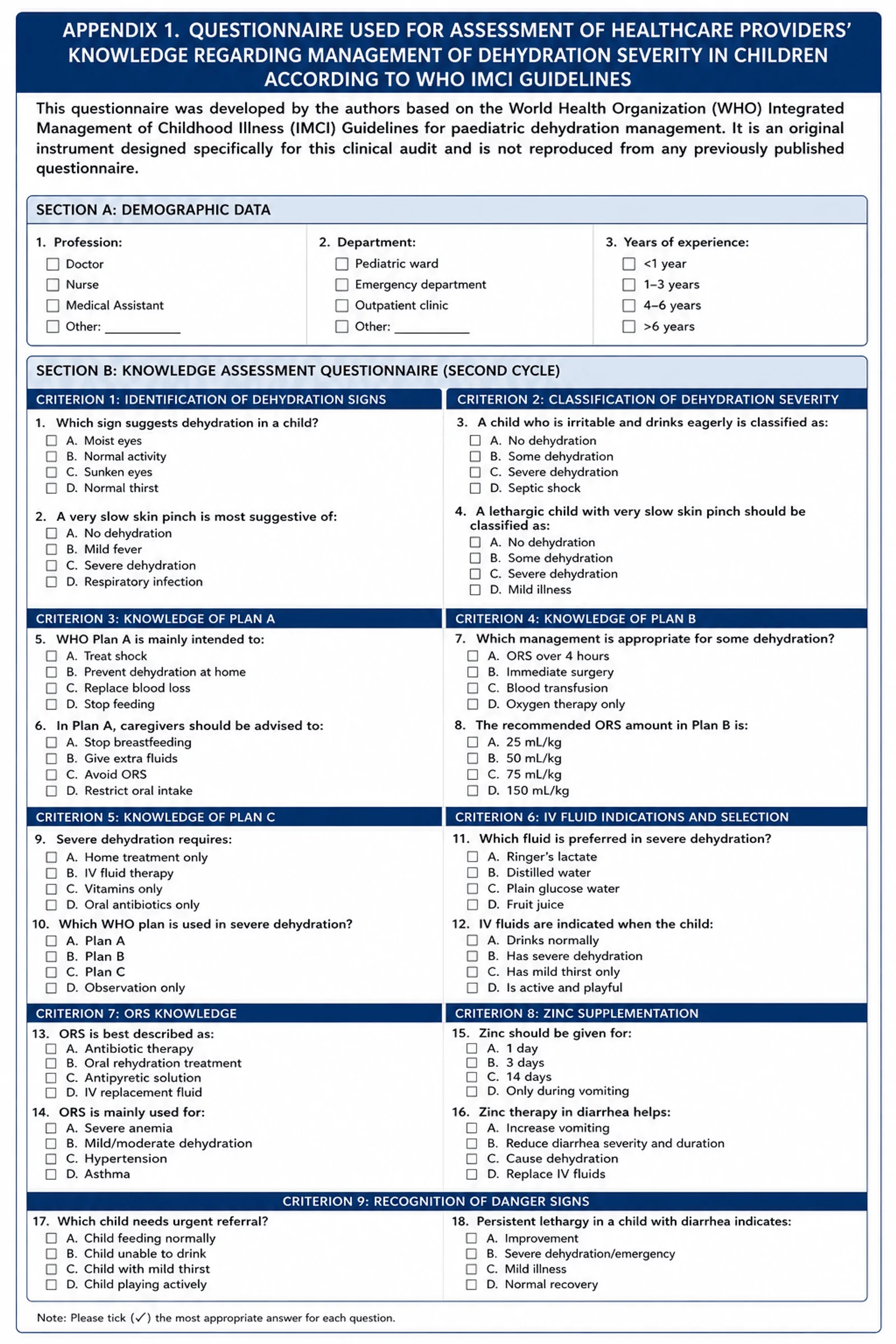
Author-developed questionnaire used to assess healthcare providers’ knowledge of paediatric dehydration management according to the World Health Organization (WHO) Integrated Management of Childhood Illness (IMCI) guidelines. Questionnaire used during both audit cycles to assess healthcare providers' knowledge of paediatric dehydration management. The questionnaire was developed by the authors based on the recommendations of the World Health Organization (WHO) Integrated Management of Childhood Illness (IMCI) guidelines and the WHO Pocket Book of Hospital Care for Children [1,4]. It was designed specifically for this clinical audit and was not adapted or reproduced from any previously published questionnaire. The same questionnaire was administered during both audit cycles to enable direct comparison of participants' knowledge before and after the educational intervention.
